# 4-Nitro­phenyl *N*-(2-sulfamoylphen­yl)carbamate

**DOI:** 10.1107/S1600536813003310

**Published:** 2013-02-06

**Authors:** Wenying Yu, Chenglong Li

**Affiliations:** a Division of Medicinal Chemistry and Pharmacognosy, College of Pharmacy, The Ohio State University, Columbus, OH 43210, USA

## Abstract

In the title mol­ecule, C_13_H_11_N_3_O_6_S, the dihedral angle between the benzene rings is 35.52 (8)°. An intra­molecular N—H⋯O hydrogen bond forms an *S*(6) ring. In the crystal, mol­ecules are linked *via* N—H⋯O hydrogen bonds into chains along [101] incorporating *R*_2_^2^(8) and *R*_2_^2^(16) rings.

## Related literature

For the synthesis, see: Mallakpour & Rafiee (2007 ▶). For hydrogen-bond graph-set motifs, see: Bernstein *et al.* (1995 ▶).
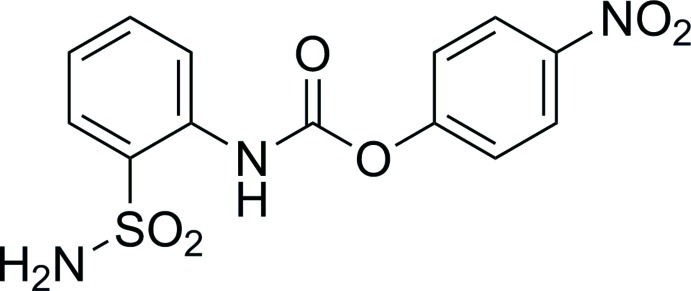


## Experimental

### 

#### Crystal data

C_13_H_11_N_3_O_6_S
*M* *_r_* = 337.31
Triclinic, 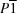

*a* = 8.2730 (2) Å
*b* = 8.4881 (2) Å
*c* = 10.4288 (2) Å
α = 95.178 (1)°
β = 103.507 (1)°
γ = 94.109 (1)°
*V* = 705.91 (3) Å^3^
*Z* = 2
Mo *K*α radiation
μ = 0.27 mm^−1^
*T* = 180 K
0.27 × 0.27 × 0.12 mm

#### Data collection

Nonius KappaCCD diffractometer
21157 measured reflections
3227 independent reflections
2473 reflections with *I* > 2σ(*I*)
*R*_int_ = 0.037

#### Refinement

*R*[*F* ^2^ > 2σ(*F* ^2^)] = 0.037
*wR*(*F* ^2^) = 0.098
*S* = 1.05
3227 reflections
220 parameters
H atoms treated by a mixture of independent and constrained refinement
Δρ_max_ = 0.23 e Å^−3^
Δρ_min_ = −0.40 e Å^−3^

### 

Data collection: *COLLECT* (Nonius, 2000 ▶); cell refinement: *SCALEPACK* (Otwinowski & Minor, 1997 ▶); data reduction: *DENZO* (Otwinowski & Minor, 1997 ▶) and *SCALEPACK*; program(s) used to solve structure: *SHELXS97* (Sheldrick, 2008 ▶); program(s) used to refine structure: *SHELXL97* (Sheldrick, 2008 ▶); molecular graphics: *ORTEP-3 for Windows* (Farrugia, 2012 ▶); software used to prepare material for publication: *WinGX* (Farrugia, 2012 ▶).

## Supplementary Material

Crystal structure: contains datablock(s) global, I. DOI: 10.1107/S1600536813003310/lh5580sup1.cif

Structure factors: contains datablock(s) I. DOI: 10.1107/S1600536813003310/lh5580Isup2.hkl

Supplementary material file. DOI: 10.1107/S1600536813003310/lh5580Isup3.cml

Additional supplementary materials:  crystallographic information; 3D view; checkCIF report

## Figures and Tables

**Table 1:** Hydrogen-bond geometry (Å, °)

*D*—H⋯*A*	*D*—H	H⋯*A*	*D*⋯*A*	*D*—H⋯*A*
N1—H1*N*1⋯O3	0.802 (19)	2.02 (2)	2.6962 (18)	142.4 (18)
N2—H1*N*2⋯O1^i^	0.83 (2)	2.15 (2)	2.975 (2)	175.1 (19)
N2—H2*N*2⋯O4^ii^	0.89 (2)	2.10 (2)	2.967 (2)	165 (2)

Symmetry codes: (i) 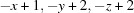; (ii) 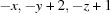.

**Fig. 1.**
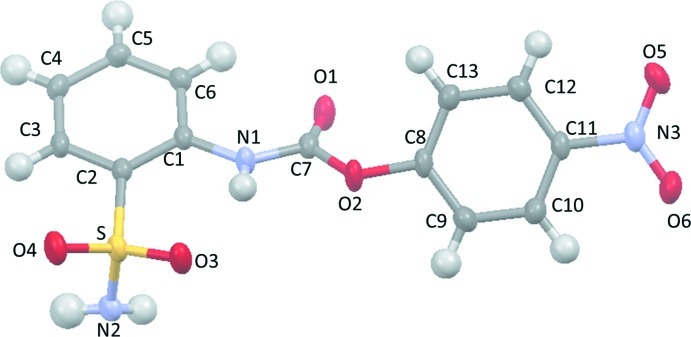
The molecular structure of the title compound with displacement ellipsoids drawn at the 50% probability level (arbitrary spheres for the H atoms).

**Fig. 2.**
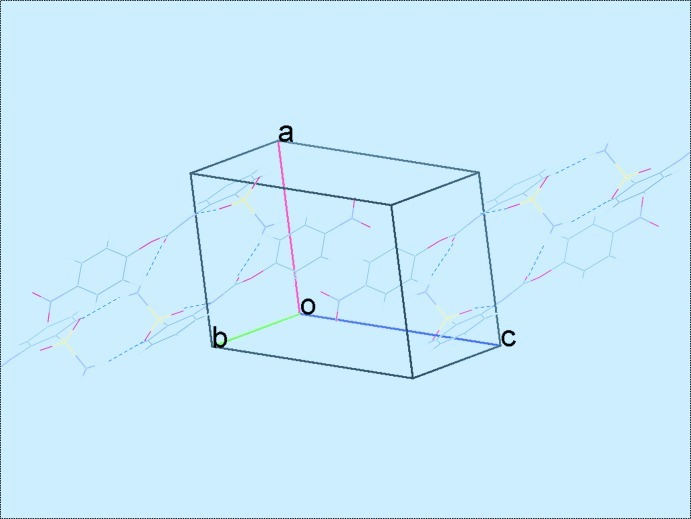
Partial crystal packing of the title compound showing the hydrogen bonds as dashed lines.

## supplementary crystallographic information

### Comment

The title compound (I) is an important intermediate in drug discovery. It was obtained by reacting 2-aminobenzenesulfonamide with 4-nitrophenyl carbonochloridate through a modified procedure by Mallakpour & Rafiee (2007). The molecular structure of the title compound is shown in Fig. 1. The dihedral angle between the two benzene rings (C1-C6 and C8-C13) is 35.52 (8)°. In the crystal, molecules are linked *via* N—H···O hydrogen bonds (Fig.2) into one-dimensional chains along [101], incorporating R^2^_2_(8) and R^2^_2_(16) rings (Bernstein *et al.*, 1995). There is also an intramolecular N—H···O hydrogen bond forming an S(6) ring.

### Experimental

All chemicals were obtained from commercial sources and used directly without further purification. 2-Aminobenzenesulfonamide (0.72 g, 1 mmol) was dissolved in 10 ml of dry tetrahydrofuran/ dichloromethane (1:1 *v*/*v*), then cooled to 273K. 4-Nitrophenyl carbonochloridate (0.2 g, 1 mmol) was added to the solution in a round-bottom flask, followed by triethylamine (0.14 ml, 1 mmol). The solution was stirred for 1 h at the same temperature and then 2h at room temperature. White solid powder precipitated out (0.307 g, yield: 91%). This was was filtered off, washed with distilled water and dried over anhydrous Na_2_SO_4_. It was characterized by its mass spectrum to be the title compound (I). Colorless plate crystals suitable for X-ray diffraction analysis were grown from a co-solvent system methanol/dichloromethane (1:20 *v*/*v*) solution by slow evaporation at 277K for a week.

### Refinement

H atoms bonded to C atoms were located in difference Fourier maps and were subsequently placed in idealized positions with C—H distances of 0.95Å. They were included in the refinemnt in a riding-motion approxmation with U_iso_(H) = 1.2U_eq_(C). H atoms bonded to N atoms were refined independently with isotropic displacement parameters.

### Crystal data

**Table:**

C_13_H_11_N_3_O_6_S	*Z* = 2
*M**_r_* = 337.31	*F*(000) = 348
Triclinic, *P*1	*D*_x_ = 1.587 Mg m^−^^3^
Hall symbol: -P 1	Mo *K*α radiation, λ = 0.71073 Å
*a* = 8.2730 (2) Å	Cell parameters from 3223 reflections
*b* = 8.4881 (2) Å	θ = 2.0–27.5°
*c* = 10.4288 (2) Å	µ = 0.27 mm^−^^1^
α = 95.178 (1)°	*T* = 180 K
β = 103.507 (1)°	Plate, colourless
γ = 94.109 (1)°	0.27 × 0.27 × 0.12 mm
*V* = 705.91 (3) Å^3^	

### Data collection

**Table:**

Nonius KappaCCD diffractometer	2473 reflections with *I* > 2σ(*I*)
Radiation source: Enraf Nonius FR590	*R*_int_ = 0.037
Graphite monochromator	θ_max_ = 27.5°, θ_min_ = 2.4°
Detector resolution: 9 pixels mm^-1^	*h* = −10→10
φ and ω scans	*k* = −11→11
21157 measured reflections	*l* = −13→13
3227 independent reflections	

### Refinement

**Table:**

Refinement on *F*^2^	Primary atom site location: structure-invariant direct methods
Least-squares matrix: full	Secondary atom site location: difference Fourier map
*R*[*F*^2^ > 2σ(*F*^2^)] = 0.037	Hydrogen site location: inferred from neighbouring sites
*wR*(*F*^2^) = 0.098	H atoms treated by a mixture of independent and constrained refinement
*S* = 1.05	*w* = 1/[σ^2^(*F*_o_^2^) + (0.051*P*)^2^ + 0.1167*P*] where *P* = (*F*_o_^2^ + 2*F*_c_^2^)/3
3227 reflections	(Δ/σ)_max_ = 0.001
220 parameters	Δρ_max_ = 0.23 e Å^−^^3^
0 restraints	Δρ_min_ = −0.40 e Å^−^^3^

### Special details

**Table:**

Experimental. All work was done at 180 K using an Oxford Cryosystems Cryostream Cooler.The data collection strategy was set up to measure a hemisphere of reciprocal space with a redundancy factor of 3.6, which means that 90% of these reflections were measured at least 3.6 times. Phi and omega scans with a frame width of 2.0 degrees were used. Data integration was done with *DENZO*, and scaling and merging of the data was done with *SCALEPACK*.
Geometry. All s.u.'s (except the s.u. in the dihedral angle between two l.s. planes) are estimated using the full covariance matrix. The cell s.u.'s are taken into account individually in the estimation of s.u.'s in distances, angles and torsion angles; correlations between s.u.'s in cell parameters are only used when they are defined by crystal symmetry. An approximate (isotropic) treatment of cell s.u.'s is used for estimating s.u.'s involving l.s. planes.
Refinement. The hydrogen atoms bonded to the nitrogen atoms were located on difference electron density maps, added to the model at these positions and refined isotropically. All three N—H groups are involved in intra and intermolecular hydrogen bonds with the oxygen atoms bonded to the *S* atom and with the oxygen atom of the carbonyl group. The rest of the hydrogen atoms were included in the model at calculated positions using a riding model with U(H) = 1.2**U*_eq_(bonded atom).Refinement of *F*^2^ against ALL reflections. The weighted *R*-factor *wR* and goodness of fit *S* are based on *F*^2^, conventional *R*-factors *R* are based on *F*, with *F* set to zero for negative *F*^2^. The threshold expression of *F*^2^ > 2σ(*F*^2^) is used only for calculating *R*-factors(gt) *etc*. and is not relevant to the choice of reflections for refinement. *R*-factors based on *F*^2^ are statistically about twice as large as those based on *F*, and *R*- factors based on ALL data will be even larger.

### Fractional atomic coordinates and isotropic or equivalent isotropic displacement parameters (Å^2^)

**Table:**

	*x*	*y*	*z*	*U*_iso_*/*U*_eq_	
C1	0.18448 (19)	1.06402 (18)	0.95047 (15)	0.0243 (3)	
C2	0.09787 (19)	1.05966 (18)	0.81639 (15)	0.0231 (3)	
C3	0.0295 (2)	1.19437 (19)	0.76876 (16)	0.0283 (4)	
H3	−0.0294	1.1901	0.6785	0.034*	
C4	0.0464 (2)	1.3342 (2)	0.85148 (17)	0.0333 (4)	
H4	−0.0004	1.4259	0.8186	0.04*	
C5	0.1322 (2)	1.3391 (2)	0.98272 (17)	0.0339 (4)	
H5	0.1446	1.4352	1.0398	0.041*	
C6	0.2001 (2)	1.2067 (2)	1.03241 (16)	0.0314 (4)	
H6	0.258	1.2127	1.1231	0.038*	
C7	0.3599 (2)	0.91756 (19)	1.11483 (16)	0.0261 (4)	
C8	0.4744 (2)	0.71841 (18)	1.24599 (16)	0.0257 (4)	
C9	0.5823 (2)	0.6064 (2)	1.22946 (17)	0.0318 (4)	
H9	0.5904	0.568	1.143	0.038*	
C10	0.6788 (2)	0.5501 (2)	1.33996 (16)	0.0304 (4)	
H10	0.754	0.4727	1.3309	0.036*	
C11	0.66298 (19)	0.60948 (18)	1.46359 (16)	0.0246 (3)	
C12	0.5552 (2)	0.72220 (18)	1.48113 (16)	0.0269 (4)	
H12	0.5475	0.761	1.5676	0.032*	
C13	0.4586 (2)	0.77744 (19)	1.37010 (16)	0.0278 (4)	
H13	0.3829	0.8544	1.379	0.033*	
N1	0.24540 (18)	0.92648 (17)	1.00104 (14)	0.0282 (3)	
H1N1	0.211 (2)	0.845 (2)	0.9546 (19)	0.033 (5)*	
N2	0.2259 (2)	0.92531 (18)	0.61879 (15)	0.0294 (3)	
H1N2	0.322 (3)	0.939 (2)	0.668 (2)	0.039 (6)*	
H2N2	0.197 (3)	0.993 (3)	0.559 (2)	0.053 (6)*	
N3	0.76147 (17)	0.54610 (16)	1.58042 (13)	0.0288 (3)	
O1	0.44001 (14)	1.02495 (13)	1.19174 (12)	0.0354 (3)	
O2	0.37252 (14)	0.75997 (13)	1.12918 (11)	0.0314 (3)	
O3	0.11646 (15)	0.75518 (13)	0.76379 (11)	0.0333 (3)	
O4	−0.07382 (14)	0.89165 (14)	0.60048 (11)	0.0333 (3)	
O5	0.73699 (16)	0.58990 (14)	1.68935 (11)	0.0365 (3)	
O6	0.86034 (16)	0.45021 (16)	1.56352 (13)	0.0425 (3)	
S	0.08224 (5)	0.89358 (5)	0.69623 (4)	0.02568 (13)	

### Atomic displacement parameters (Å^2^)

**Table:**

	*U*^11^	*U*^22^	*U*^33^	*U*^12^	*U*^13^	*U*^23^
C1	0.0236 (8)	0.0292 (8)	0.0209 (8)	0.0074 (6)	0.0035 (6)	0.0071 (6)
C2	0.0215 (8)	0.0268 (8)	0.0206 (8)	0.0048 (6)	0.0025 (6)	0.0049 (6)
C3	0.0275 (9)	0.0360 (9)	0.0221 (8)	0.0121 (7)	0.0025 (7)	0.0089 (7)
C4	0.0383 (10)	0.0341 (9)	0.0305 (9)	0.0182 (8)	0.0075 (8)	0.0093 (7)
C5	0.0412 (10)	0.0333 (9)	0.0279 (9)	0.0159 (8)	0.0070 (8)	0.0007 (7)
C6	0.0363 (10)	0.0379 (10)	0.0199 (8)	0.0139 (8)	0.0033 (7)	0.0035 (7)
C7	0.0253 (8)	0.0294 (9)	0.0239 (8)	0.0067 (7)	0.0037 (7)	0.0077 (7)
C8	0.0259 (8)	0.0249 (8)	0.0236 (8)	0.0019 (7)	−0.0017 (7)	0.0091 (7)
C9	0.0378 (10)	0.0363 (10)	0.0220 (8)	0.0094 (8)	0.0061 (7)	0.0054 (7)
C10	0.0314 (9)	0.0331 (9)	0.0282 (9)	0.0111 (7)	0.0061 (7)	0.0077 (7)
C11	0.0233 (8)	0.0249 (8)	0.0231 (8)	0.0003 (6)	−0.0005 (6)	0.0083 (6)
C12	0.0309 (9)	0.0264 (8)	0.0221 (8)	0.0019 (7)	0.0041 (7)	0.0028 (6)
C13	0.0280 (9)	0.0263 (8)	0.0291 (9)	0.0070 (7)	0.0042 (7)	0.0058 (7)
N1	0.0338 (8)	0.0256 (8)	0.0213 (7)	0.0075 (6)	−0.0031 (6)	0.0041 (6)
N2	0.0271 (8)	0.0359 (8)	0.0218 (7)	0.0047 (6)	−0.0002 (6)	0.0005 (6)
N3	0.0300 (8)	0.0277 (7)	0.0254 (8)	0.0004 (6)	−0.0011 (6)	0.0082 (6)
O1	0.0332 (7)	0.0303 (6)	0.0346 (7)	0.0026 (5)	−0.0094 (6)	0.0073 (5)
O2	0.0356 (7)	0.0277 (6)	0.0251 (6)	0.0060 (5)	−0.0068 (5)	0.0080 (5)
O3	0.0436 (7)	0.0251 (6)	0.0262 (6)	0.0035 (5)	−0.0025 (5)	0.0055 (5)
O4	0.0273 (6)	0.0382 (7)	0.0276 (6)	−0.0025 (5)	−0.0065 (5)	0.0071 (5)
O5	0.0470 (8)	0.0380 (7)	0.0210 (6)	0.0003 (6)	0.0014 (6)	0.0053 (5)
O6	0.0425 (8)	0.0487 (8)	0.0364 (7)	0.0199 (6)	0.0018 (6)	0.0135 (6)
S	0.0258 (2)	0.0270 (2)	0.0203 (2)	0.00131 (16)	−0.00231 (16)	0.00350 (16)

### Geometric parameters (Å, º)

**Table:**

C1—C6	1.397 (2)	C9—C10	1.385 (2)
C1—N1	1.402 (2)	C9—H9	0.95
C1—C2	1.410 (2)	C10—C11	1.380 (2)
C2—C3	1.392 (2)	C10—H10	0.95
C2—S	1.7758 (16)	C11—C12	1.382 (2)
C3—C4	1.381 (2)	C11—N3	1.468 (2)
C3—H3	0.95	C12—C13	1.385 (2)
C4—C5	1.382 (2)	C12—H12	0.95
C4—H4	0.95	C13—H13	0.95
C5—C6	1.381 (2)	N1—H1N1	0.802 (19)
C5—H5	0.95	N2—S	1.6063 (16)
C6—H6	0.95	N2—H1N2	0.83 (2)
C7—O1	1.2028 (19)	N2—H2N2	0.89 (2)
C7—N1	1.346 (2)	N3—O6	1.2246 (17)
C7—O2	1.3680 (19)	N3—O5	1.2296 (18)
C8—C9	1.376 (2)	O3—S	1.4354 (12)
C8—C13	1.384 (2)	O4—S	1.4339 (11)
C8—O2	1.3999 (18)		
			
C6—C1—N1	121.20 (14)	C11—C10—H10	120.9
C6—C1—C2	118.12 (14)	C9—C10—H10	120.9
N1—C1—C2	120.60 (14)	C10—C11—C12	122.69 (15)
C3—C2—C1	120.27 (14)	C10—C11—N3	118.21 (14)
C3—C2—S	115.83 (12)	C12—C11—N3	119.07 (15)
C1—C2—S	123.72 (12)	C11—C12—C13	118.70 (15)
C4—C3—C2	120.66 (15)	C11—C12—H12	120.7
C4—C3—H3	119.7	C13—C12—H12	120.7
C2—C3—H3	119.7	C8—C13—C12	118.75 (15)
C3—C4—C5	119.19 (15)	C8—C13—H13	120.6
C3—C4—H4	120.4	C12—C13—H13	120.6
C5—C4—H4	120.4	C7—N1—C1	127.37 (15)
C6—C5—C4	121.18 (16)	C7—N1—H1N1	117.1 (14)
C6—C5—H5	119.4	C1—N1—H1N1	115.5 (14)
C4—C5—H5	119.4	S—N2—H1N2	113.9 (14)
C5—C6—C1	120.58 (15)	S—N2—H2N2	111.1 (14)
C5—C6—H6	119.7	H1N2—N2—H2N2	118 (2)
C1—C6—H6	119.7	O6—N3—O5	123.82 (14)
O1—C7—N1	128.09 (15)	O6—N3—C11	118.24 (14)
O1—C7—O2	124.49 (14)	O5—N3—C11	117.92 (13)
N1—C7—O2	107.42 (14)	C7—O2—C8	118.65 (12)
C9—C8—C13	122.16 (14)	O4—S—O3	118.58 (7)
C9—C8—O2	115.74 (14)	O4—S—N2	106.39 (8)
C13—C8—O2	121.93 (14)	O3—S—N2	107.65 (8)
C8—C9—C10	119.44 (15)	O4—S—C2	107.69 (7)
C8—C9—H9	120.3	O3—S—C2	108.65 (7)
C10—C9—H9	120.3	N2—S—C2	107.39 (8)
C11—C10—C9	118.26 (15)		
			
C6—C1—C2—C3	−0.5 (2)	C11—C12—C13—C8	−0.4 (2)
N1—C1—C2—C3	176.32 (15)	O1—C7—N1—C1	−4.9 (3)
C6—C1—C2—S	174.44 (13)	O2—C7—N1—C1	175.73 (15)
N1—C1—C2—S	−8.8 (2)	C6—C1—N1—C7	−19.4 (3)
C1—C2—C3—C4	0.5 (3)	C2—C1—N1—C7	163.86 (16)
S—C2—C3—C4	−174.82 (13)	C10—C11—N3—O6	4.1 (2)
C2—C3—C4—C5	−0.1 (3)	C12—C11—N3—O6	−177.65 (14)
C3—C4—C5—C6	−0.4 (3)	C10—C11—N3—O5	−174.26 (15)
C4—C5—C6—C1	0.4 (3)	C12—C11—N3—O5	3.9 (2)
N1—C1—C6—C5	−176.73 (16)	O1—C7—O2—C8	5.7 (2)
C2—C1—C6—C5	0.1 (3)	N1—C7—O2—C8	−174.90 (14)
C13—C8—C9—C10	−0.1 (3)	C9—C8—O2—C7	−132.47 (16)
O2—C8—C9—C10	−175.35 (15)	C13—C8—O2—C7	52.2 (2)
C8—C9—C10—C11	−0.1 (3)	C3—C2—S—O4	−35.25 (15)
C9—C10—C11—C12	0.0 (3)	C1—C2—S—O4	149.62 (14)
C9—C10—C11—N3	178.11 (15)	C3—C2—S—O3	−164.86 (12)
C10—C11—C12—C13	0.3 (3)	C1—C2—S—O3	20.01 (16)
N3—C11—C12—C13	−177.85 (14)	C3—C2—S—N2	78.97 (14)
C9—C8—C13—C12	0.3 (3)	C1—C2—S—N2	−96.16 (15)
O2—C8—C13—C12	175.31 (14)		

### Hydrogen-bond geometry (Å, º)

**Table:**

*D*—H···*A*	*D*—H	H···*A*	*D*···*A*	*D*—H···*A*
N1—H1*N*1···O3	0.802 (19)	2.02 (2)	2.6962 (18)	142.4 (18)
N1—H1*N*1···S	0.802 (19)	2.736 (19)	3.1283 (14)	112.2 (15)
N2—H1*N*2···O1^i^	0.83 (2)	2.15 (2)	2.975 (2)	175.1 (19)
N2—H2*N*2···O4^ii^	0.89 (2)	2.10 (2)	2.967 (2)	165 (2)

Symmetry codes: (i) −*x*+1, −*y*+2, −*z*+2; (ii) −*x*, −*y*+2, −*z*+1.
